# Lipohypertrophy and metabolic disorders in HIV patients on antiretroviral therapy: a systematic multidisciplinary clinical approach

**DOI:** 10.7448/IAS.17.4.19559

**Published:** 2014-11-02

**Authors:** Delphine Sculier, Laurence Toutous-Trellu, Charlotte Verolet, Nicolas Matthes, Thanh Lecompte, Alexandra Calmy

**Affiliations:** 1 Infectious Diseases, University Hospitals of Geneva, Geneva, Switzerland; 2 Dermatology and Venerology, University Hospitals of Geneva, Geneva, Switzerland; 3 Pediatrics, University Hospitals of Geneva, Geneva, Switzerland

## Abstract

**Introduction:**

Morphological and metabolic complications in HIV patients on antiretroviral therapy remain a challenge. While new cases of lipoatrophy (LA) disappear, irreducible central lipohypertrophy (LH) and metabolic complications require highly specialized management. We described a day hospital dedicated to lipodystrophy (LD) and metabolic disorders in HIV patients on treatment in Geneva, Switzerland, with a focus on LH.

**Materials and Methods:**

The “Groupe Lipo & Metabolism” is a multidisciplinary consultation where patients undergo a standard evaluation including questionnaire, physical examination, dual-energy x-ray absorptiometry (DEXA) and L5-level CT scans, blood tests and consultations with various specialists. Based on prospectively maintained data, we describe clinical, biological and radiological characteristics of patients ≥18 years who attended the consultation between 2008 and 2013. We defined LH by CT scan, the gold standard method, as abdominal visceral adipose tissue (VAT) ≥130 cm^2^, value associated with increased risk of cardiovascular event.

**Results:**

A total of 195 patients attended the consultation during study period. Reasons for referral included LH in 28.3%, LA in 25% and mixed syndrome in 15.5% of cases. Metabolic disorders accounted for 19% of referrals with or without LD features. Among patients with a CT scan performed (n=183), 46 (25%) had LH with a VAT ≥130 cm^2^. In this population, mean age was 49.1 years and 53.6% were male. HIV viral load was <50 cp/ml in 87% of patients. Mean body mass index was 24.6 kg/m^2^. Mean waist to hip ratio (WHR) was 0.98 for males and 0.89 for females. A total of 9.8%, 29.5% and 35% of patients had abnormal levels of total cholesterol (≥6.5 mmol/L), triglycerides (≥2.0 mmol/L) and HDL cholesterol (≤1.0 mmol/L), respectively. Mean fasting glycaemia was 5.7 mmol/L and HbA1c was >6% in 10.5% of patients. Vitamin-D level was <75 nmol/L in 70.7% of patients. Respectively 31.2% and 12.1% of patients had osteopenia and osteoporosis on the spine and 44.8% and 6.6% on the hip neck. Factors associated with a VAT≥130 cm^2^ included male gender (OR 3.7 [95% CI 1.7–8.2] p<0.001), triglycerides ≥2 mmol/L (OR 2.6 [95% CI 1.3–5.4] P<0.01) and increase in BMI category (OR 1.8 [95% CI 1.2–2.8] p<0.01).

**Conclusions:**

Lipohypertrophy is a prevalent feature of fat redistribution among HIV patients on treatment. Risk factors for LH include male gender, dyslipidemia and overweight. Glucose impairment and bone disorders are also common. A multidisciplinary approach is important to identify and promptly address these disorders.

**Acknowledgments:**

The “Groupe Lipo & Metabolism” team.

